# Combining graph neural networks and spatio-temporal disease models to improve the prediction of weekly COVID-19 cases in Germany

**DOI:** 10.1038/s41598-022-07757-5

**Published:** 2022-03-10

**Authors:** Cornelius Fritz, Emilio Dorigatti, David Rügamer

**Affiliations:** 1 Department of Statistics, Ludwig Maximilian Universität, München, Germany; 2 Institute for Computational Biology, Helmholtz Zentrum München—German Research Center for Environmental Health, Neuherberg, Germany

**Keywords:** Computer science, Statistics, Machine learning, Statistical methods

## Abstract

During 2020, the infection rate of COVID-19 has been investigated by many scholars from different research fields. In this context, reliable and interpretable forecasts of disease incidents are a vital tool for policymakers to manage healthcare resources. In this context, several experts have called for the necessity to account for human mobility to explain the spread of COVID-19. Existing approaches often apply standard models of the respective research field, frequently restricting modeling possibilities. For instance, most statistical or epidemiological models cannot directly incorporate unstructured data sources, including relational data that may encode human mobility. In contrast, machine learning approaches may yield better predictions by exploiting these data structures yet lack intuitive interpretability as they are often categorized as black-box models. We propose a combination of both research directions and present a multimodal learning framework that amalgamates statistical regression and machine learning models for predicting local COVID-19 cases in Germany. Results and implications: the novel approach introduced enables the use of a richer collection of data types, including mobility flows and colocation probabilities, and yields the lowest mean squared error scores throughout the observational period in the reported benchmark study. The results corroborate that during most of the observational period more dispersed meeting patterns and a lower percentage of people staying put are associated with higher infection rates. Moreover, the analysis underpins the necessity of including mobility data and showcases the flexibility and interpretability of the proposed approach.

## Introduction

In December 2019, the region of Wuhan, China, experienced an outbreak of a novel coronavirus, COVID-19, initially infecting around 40 people^1^. The disease quickly spread throughout the world, because ill people are already infectious in the pre-symptomatic stage of the disease and transmission occurs through the exchange of virus-containing droplets or expiratory particles^2^. Consequentially, the World Health Organization declared COVID-19 a pandemic in March 2020, and more than 66 million infections and 1.5 million deaths were registered worldwide by the end of that year^3^.

Given this development, it was repeatedly pondered if and how mathematical modeling could help to contain the COVID-19 crisis^4,5^. We argue that data science and machine learning can provide urgently needed tools to doctors and policymakers in various applications. For instance, model-assisted identification and localization of COVID-19 in chest X-rays can support doctors in achieving correct and precise diagnoses^6^. Meanwhile, policymakers benefit from studies determining and evaluating specific policies^2^. In one noteworthy example, it was possible to quantify to what extend targeted non-pharmaceutical interventions aided in eliminating the exponential growth rate of COVID-19 cases^7^. This work is often quoted as the main driver of the social distancing measures implemented in the UK, thus allowing the British government to pursue evidence-based containment strategies. Other works use mobility data provided by multiple technology companies, such as Apple^8^, Google^9^, and Facebook^10^. To name a few examples^11^, assess the similarities between the spread of COVID-19, community activity as measured by data provided by Apple, and financial performance. Heterogeneities in the welfare cost of staying at home between different census block groups, on the other hand, are uncovered by relying on mobility data from Google in^12^. Finally^13^, utilize data from Facebook to gauge social and geographic spillover effects from loosening shelter-in-place orders.

In order to adequately evaluate the role of specific policies and implement a successful containment strategy, a robust and interpretable forecast of the pandemic’s state into the future is necessary. Among other purposes, this endeavor allows authorities to better manage healthcare resources such as hospital beds, respirators, and vaccines. The corresponding modeling task is broad and can be tackled at various levels of spatio-temporal granularity. While some proposals operate on daily country-level data^14^, others are designed to provide local predictions^15^. From a methodological point of view, most of these approaches are influenced by either epidemiology, time series analysis, regression models, machine or deep learning. However, we argue that the most promising approaches combine ideas from seemingly distant research areas such that new types of data can be used. Thereby, one can bypass the restrictions of simpler models and improve the forecasting performance while also benefiting from the merits of each respective idea. In addition, allowing for the inclusion of novel data modalities in some of the more traditional approaches may further improve models. This was highlighted in previous works^16^ that included non-standard data sources, e.g., aggregated contact patterns obtained from mobile phones or behavioral data, into the analysis to help in understanding and fighting COVID-19.

### Hybrid modeling approaches

Examples of these types of hybrid models are scattered throughout the literature. In^15^ combined a mechanistic metapopulation commonly used in epidemiology with clustering and data augmentation techniques from machine learning to improve their forecasting performance. For this endeavor, additional data sources, such as news reports and internet search activity, were leveraged to inform the global epidemic and mobility model, an epidemiological model already successfully applied to the spread of the Zika virus^17^. In another notable application^18^, enriched a relatively simple metapopulation model with mobility flows to numerous points of interest. Subsequently, this model was used to predict the effect of reopening after a specific type of lock-down through counterfactual analysis. With the help of Facebook^19^, utilized mobility and aggregated friendship networks to discover how these networks drove the infection rates on the local level of federal districts in Germany. In this context, another route to accommodate such network data is through graph neural networks (GNNs). These techniques build on the intuitive idea of message passing between nodes^20^ and have recently attracted a lot of attention in the deep learning community^21^. Among the wide range of use cases of GNNs are node classification as well as forecasting^21,22^.

### Applications of graph neural networks to COVID-19 data

In an early example of such works^23^, constructed a graph whose edges encode mobility data for a given time point collected from Facebook. Their approach exploited a long short-term memory architecture to aggregate latent district features obtained from several graph convolutional layers and transfer learning to account for the asynchronous nature of outbreaks across borders. In a comparable proposal^24^, employed a GNN to encode spatial neighborhoods and a recurrent neural network (RNN) to aggregate information in the temporal domain. Through a novel loss function, they simultaneously penalized the squared error of the predicted infected and recovered cases as well as the long-term error governed by the transmission and recovery rates within traditional Susceptible–Infectious–Recovered models. Contrasting these approaches^25^, proposed a RNN to derive latent features for each location, hence they constructed a graph whose edge weights were given by a self-attention layer. Instead of using a RNN^26^, constructed a spatio-temporal graph by creating an augmented spatial graph that included all observed instances of the observed network side-by-side and enabled temporal dependencies by connecting each location with the corresponding node in previous days.

### Contribution

Against this background, we propose a novel fusion approach that directly combines dyadic mobility and connectedness data derived from the online platform Facebook with structural and spatial information of Germany’s cities and districts. In contrast to^19^, the network learns each district’s embedding in an end-to-end fashion, thus there is no need for a separate pre-processing step. With this, we heed recent calls such as^16,27^ highlighting the need for more flexible and hybrid approaches taking also dyadic sources of information into account. From a methodological point of view, we make this possible by combining graph neural networks with epidemiological models^28,29^ to simultaneously account for network-valued data and tabular data. We further provide comparisons, sanity checks as well as uncertainty quantification to investigate the reliability of the presented model. When applying the model, we provide forecasts of weekly COVID-19 cases with disease onset at the local level of 401 federal districts in Germany as provided by the Robert–Koch Institute.

## Data

Concerning the data sources, we distinguish between infection and Facebook data on human mobility and connectedness. While the infection data are time series solely utilized in the model’s structured and target component, most network data is used directly in the GNN module. To allow for sanity checks and interpretable coefficients, the networks are also transformed onto the units where we measured the time series by calculating specific structural characteristics from the networks following^19^.

### Time series of daily COVID-19 infections

**Figure 1:**
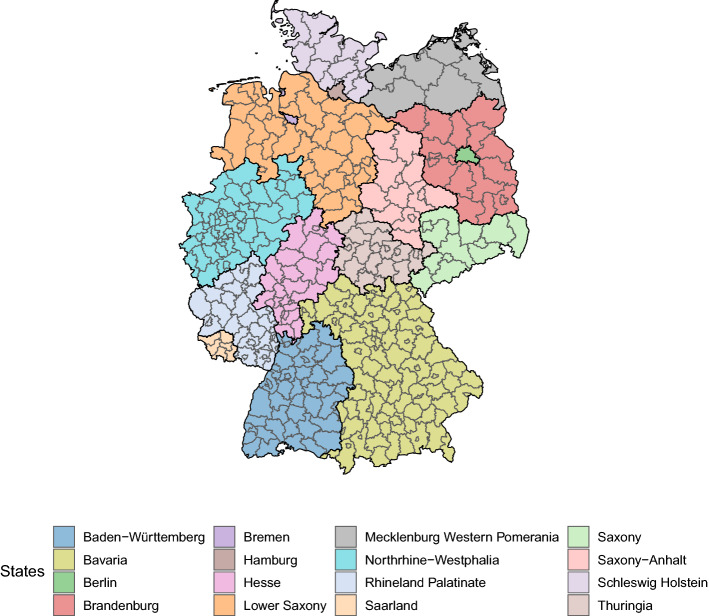
Map of all German federal districts color-coded according to the state they are allocated. The thick black lines indicate borders between federal states, while the thinner grey borders separate federal districts, which is the spatial unit on which the infection data are available. The map was created using the software R^30^ with packages sf^31^ and ggplot2^32^.

For the analysis, we use current data on the state of the pandemic in Germany provided by the Robert–Koch Institute^33^. This database constitutes the empirical basis on which most non-pharmaceutical interventions in Germany were carried and includes, among other information, the number of people with symptom onset and registered cases of COVID-19 grouped by age, gender, and federal district (NUTS-3 level) for each day. Figure 1 depicts the districts and how they are situated in a state. Due to the observation that mainly people aged between 15 and 59 years are active users of Facebook, we limit the analysis to the corresponding age groups, namely, people with age between 15–35 and 36–59.

As discussed in^34^, the central indicator of the infection occurrence is the number of people with disease onset on a specific day, accordingly the presented application focuses on this quantity. Due to mild cases of infection and inconsistent data collection, the data of disease onset is not known in about 30$\%$ of the cases. Therefore, missing values are imputed by learning a probabilistic model of the delay between disease onset and registration date. Eventually, we attain $y_{ig}(t)$, the infection counts of district *i* and group *g* during week *t*, by sampling from the estimated distribution of delay times. By doing that, we follow^19^ where the procedure is given in more detail. The groups *g* are elements of the Cartesian product of all possible age and gender groups. We denote all corresponding features by the vector $\varvec{x}_{ig}(t)$.

Further, we are given data on the population sizes of each group *g* and district *i* from the German Federal Statistical Office, denoted by $pop_{ig}$, based on which we compute the population density $den_{ig}$. In this setting, one can assume that not the count but rate of infections in a specific district and group carries vital information. Hence, the target we model is the corresponding rate defined by $\tilde{y}_{ig}(t) = \frac{y_{ig}(t)}{pop_{ig}}$.

### Facebook data on human mobility and connectedness

To quantify the social and mobility patterns on the regional level, we use data on friendship ties, colocation probabilities, and district-specific data on the proportion of people staying put provided by Facebook^35^. These spatial data sets were made available through the *Data for Good* program^10^ and used in several other publications, such as^19,36^. More details on the data set and information how it can be accessed is given in the Appendix. Facebook collected all data from individual mobile phone location traces of Facebook users aged over 18 that opted in the *Location History* feature on the mobile Facebook application. To preserve users’ privacy, these individual traces were aggregated using differential privacy for each of the $n = 401$ federal districts^35^. Thus the resolution of the mobility data is the same as for the infection data. Most data are measured on the dyadic level between federal districts, hence perceiving the data as spatial networks comes naturally. The nodes in these networks are, therefore, these districts. We enrich the given network data with spatial networks encoding neighboring districts and distances in kilometers, that are respectively denoted by $x^N \in \lbrace 0,1 \rbrace ^{n\times n}$ and $x^D \in \mathbb {R}_{>0}^{n\times n}$. Consecutively, each type of provided information is described in detail.

#### Co-location networks

The first type of network data that we incorporate in the forecast are co-location maps, which indicate the probability of two random people from two districts to meet one another during a given week^37^. For each week *t*, we have a co-location matrix $x^{C}(t)\in \mathbb {R}_{>0}^{n \times n}$, where the *i*, *j*-th entry $x_{ij}^{C}(t)$ gives us the probability of an arbitrary person from district *i* meeting another person from district *j*. To also incorporate such network-valued data in the structured part of the suggested framework, we transform it to tabular data. In particular, we follow^19^ and use the Gini index to measure the concentration of meeting patterns of districts. For district $i \in \lbrace 1, ..., n\rbrace$ in week *t* this index is defined as:

1 $$\begin{aligned} x_{i}^G(t) = \frac{\sum _{m, n \ne i} |x_{im}^C(t) -x_{in}^C(t) |}{2 (n-1) \sum _{j\ne i} x_{ij}^C(t) } \in [0,1]. \end{aligned}$$

Higher values of Eq. (1) for a specific district *i* in week *t* translate to a restricted meeting pattern within a district, while lower values suggest rather diffused social behavior. We additionally perform a weekly standardization of $x_{i}^G(t)$:

2 $$\begin{aligned} \tilde{x}_{i}^G(t) = \frac{x_{i}^G(t) - \hat{\mu }_{t}^G}{ \hat{\sigma }_{t}^G}, \end{aligned}$$

where $$\hat{\mu }_{t}^G = \frac{1}{n} \sum _{j = 1}^n x_{j}^G(t) $$ and $$\hat{\sigma }_{t,gini} = \sqrt{\frac{1}{n-1} \sum _{j = 1}^n \left( x_{j}^G(t) - \hat{\mu }_{t}^G\right) ^2 }$$.

#### Social connectedness network

Secondly, we quantify social connections between the districts via Facebook friendships. Utilizing information from a snapshot of all Facebook connections within Germany of April 2020, we derive the Social Connectedness Index as first introduced by^38^. This pairwise time-constant index relates to the relative friendship strength between two districts and is stored in a weighted network $x^S \in \mathbb {R}_{>0}^{n \times n}$. For $i,j \in \lbrace 1, \ldots , n \rbrace$ the entries of $x^S$ are given by:

3 $$\begin{aligned} x_{ij}^S= \frac{\# \lbrace \text {Friendship Ties between users in district }i \text {\,and\,} j \rbrace }{\# \lbrace \text {Users in district} i \rbrace \# \lbrace \text {Users in district }j \rbrace }. \end{aligned}$$

Via multidimensional scaling^39^, we can obtain two-dimensional district-specific embeddings from $x^S$, which we denote by $x_i^S$ and incorporate in the structured component.

#### Staying put

Besides, we incorporate the weekly percentage of people staying put as a measure for lockdown compliance among Facebook users. We derive the corresponding weekly district-specific measure $x_i^{SP}(t)$ by averaging daily measures provided by Facebook. In this context, an individual is defined as $\textit{staying put}$ on a specific day, when it is only observed in one $0.6km \times 0.6km$ square throughout the respective day^40^. Similar to Eq. (2), we standardize the covariate values for each week and denote the result for district *i* in week *t* by $\tilde{x}_i^{SP}(t)$ .

## Methodological background

To incorporate modeling techniques from statistics and epidemiology in a graph neural network, we use structured additive predictors that represent smooth additive effects of input features and can be represented in a neural network. For the smooth effects, we impose regularization terms to achieve smoothness.

### Distributional regression

Distributional regression is a modeling approach to define a parametric distribution $\mathcal {D}$ through its distributional parameters which in turn are modeled using *p* given input features $\varvec{x} \in \mathbb {R}^p$^41^. In contrast to other regression approaches that, e.g., only relate the mean of an outcome variable to certain features, distributional regression also accounts for the uncertainty of the data distribution, known as aleatoric uncertainty^42^. Given a parametric distributional assumption $\mathcal {D}(\theta _1,\ldots ,\theta _K)$, the model learns the distributional parameters $\varvec{\theta } = (\theta _1,\ldots ,\theta _K)^\top$ by means of feature effects. In structured additive distributional regression^43^, each distributional parameter is estimated using an additive predictor $\eta _k(\varvec{x}_k)$. In this context, $\eta _k: \mathbb {R}^{p_k} \rightarrow \mathbb {R}$ is an additive transformation of a pre-specified set of features $\varvec{x}_k \in \mathbb {R}^{p_k}$, $1\le p_k\le p$. This additive predictor is finally transformed to match the domain of the respective parameter by a monotonic and differentiable transformation function $h_k$:

4 $$\begin{aligned} \theta _k(\varvec{x}_k) = h_k\big (\eta _k(\varvec{x}_k)\big ). \end{aligned}$$

Note that the *K* parameters relating to $\mathcal {D}$ now depend on the features.

Moreover, structured additive distributional regression models allow for a great variety of feature effects in the additive predictor that are naturally interpretable^44^. Examples include linear, non-linear, or random effects of one or more features. The latter two effect types can be represented via basis functions (such as regression splines, polynomial bases or B-splines). Further examples and details can be found, e.g., in^45^. Due to the additivity of effects in $\eta _{k}$, the influence of single features can often be directly related to the mean or the variance of the modeled distribution, making the model inherently interpretable.

#### Semi-structured deep distributional regression

A recent trend is the combination of neural networks with statistical regression models in various ways. The authors of^46^, for instance, propose a wide-and-deep neural network that fuses a deep multi-layer perceptron with a generalized linear model. Combinations with other model classes, such as mixture models^47^ or transformation models^48^, have also been proposed. In this work, we make use of *semi-structured deep distributional regression* (SDDR,^49^). SDDR combines neural networks and structured additive distributional regression by embedding the statistical regression model in the neural network and ensures the identifiability of the regression model part. SDDR is a natural extension of distributional regression by extending the additive predictor ${\eta }_{k}$ of each distributional parameter with its *structured effects* to latent features, so-called *unstructured effects*, that are learned by one or more deep neural networks (DNN). An orthogonalisation cell is used to disentangle the structured model parts from the unstructured parts and to ensure the identifiability of the structured model effects.

### Graph neural networks

Graphs are a mathematical description of the entities and their relationships in a given domain and naturally arise in a variety of seemingly distant fields, for instance, biology^50^, political science^51^, and economics^52^. However, due to their non-euclidean structure, it is not straightforward to apply traditional machine learning methods to problems involving graphs, as these methods operate on vectors in $\mathbb {R}^n$ for some $n \in \mathbb {N}$^22^. Several methods have been introduced to embed graphs into low-dimensional Euclidean spaces, allowing to use the resulting vector representations for prediction tasks with traditional machine learning algorithms such as node classification and missing link prediction^21^. Inspired by the success of convolutional neural networks, comparable approaches formulated convolutions for graphs via the spectral domain relying on the convolution theorem^53^. Later versions of convolutional operators were adapted to the vertex domain through a message-passing framework^20^. Within this framework, each node is updated with the information on the feature vectors of its neighbors and the edges connected to them^21^. Following this work, more advanced neighborhood aggregation methods^54^, scalable inference^55^ and domain-specific applications^20^ have been introduced. In general, a graph neural network performs *R* rounds of message passing, after which all nodes’ latent features are combined to obtain a unified representation for the whole graph^56^, or individual nodes^57^. The latter type of representations is used to derive node-specific predictions.

Formally, a graph is a tuple $\langle V, E\rangle$ consisting of a set of vertices *V* and a set of edges $E\subseteq V\times V$. The edge connecting nodes *v* and *w* is denoted as $e_{vw}$ and can be associated with an edge-specific feature vector $\varvec{u}_{vw}\in \mathbb {R}^{d^e}$. Similarly, all nodes $v\in V$ can be associated with feature vectors $\varvec{u}_v^r\in \mathbb {R}^{d^v_r}$ that are transformed in *r* successive rounds, $1\le r\le R$. The initial feature vectors $\varvec{u}^1_v$ are given by vertex-specific information, such as the Gini index and percentage staying put defined in the previous section depending on the specific application. Following the notation in^20^, in each message-passing round *r* the features of the neighbors $N(v)\subseteq V$ of $v\in V$ and of the connecting edges are aggregated into a message $\varvec{m}_v^r\in \mathbb {R}^{d^m_r}$ through a message function $M_r$:

5 $$\begin{aligned} \varvec{m}_v^r=\sum _{w\in N(v)} M_r(\varvec{u}_v^r, \varvec{u}_w^r, \varvec{u}_{vw}) \end{aligned}$$

Note that edge features are considered to be constants in this framework. Next, an update function $U_r$ combines $\varvec{u}_v^r$ and $\varvec{m}_v^r$ to obtain the updated latent features $\varvec{u}_v^{r+1}$:

6 $$\begin{aligned} \varvec{u}_v^{r+1}=U_r(\varvec{u}_v^r, \varvec{m}_v^r) \end{aligned}$$

Taken together Eqs. (5) and (6) define a message passing round that propagates information one hop further than the previous round. For instance, one of the very first graph neural networks, namely the graph convolutional network^57^, proposed the following functions:

7 $$\begin{aligned}&M_r^{\text {GCN}}(\varvec{u}_v^r, \varvec{u}_w^r, u_{vw}) = \frac{u_{vw}}{\sqrt{d_{v}\cdot d_{w}}} \cdot \varvec{u}_w^r+\frac{u_{vv}}{|N(v)|\cdot d_v}\cdot \varvec{u}_v^r \end{aligned}$$

8 $$\begin{aligned}&U_r^{\text {GCN}}(\varvec{u}_v^r, \varvec{m}_v^r)=\varvec{\Theta }\cdot \varvec{m}_v^r, \end{aligned}$$

where $d_i=1+\sum _{j\in N(v)}u_{ji}$. In this case, edges are, however, only associated with scalar weights rather than feature vectors. We can apply multiple message passing rounds successively to diffuse information across the complete network. After *R* message passing rounds, a multilayer perceptron may be used to transform the node features into more abstract representations. Such feature vectors are called *node embeddings* and indicated with $\varvec{u}_v$.

### Ethics

The authors confirm that all experiments were performed in accordance with relevant guidelines and regulations.

## Combining network-valued and spatio-temporal disease data

**Figure 2:**
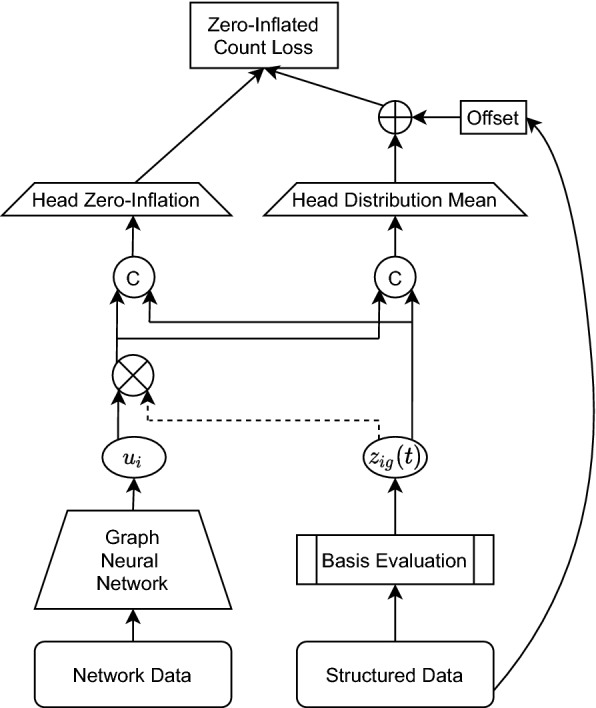
Network architecture of the proposed model. The mobility data is fed into a GNN on the bottom left to learn latent features $\varvec{u}_i$. On the bottom right side, the structured data is transformed using basis evaluation. Using the orthogonalization the learned effects $\varvec{u}_i$ are projected onto the orthogonal complement of selected parts of the basis evaluated structured features. Both parts are combined using a concatenation and fed into both a network head that learns the zero-inflation as well as a network head to learn the distribution’s mean. After adding an offset to the mean, both parts are finally combined in a distributional layer that learns the zero-inflated count distribution based on the corresponding log-likelihood

The general framework of the hybrid modeling approach to forecast weekly district-wise COVID-19 cases based on structured and unstructured data sources is depicted in Fig. 2 and fuses the interpretability of distributional regression with a GNN architecture to flexibly learn all district’s latent representation from the network data.

### Neural network formulation

#### Distributional assumption

The considered time window stretches over a low-infection phase during which 20–30% of the districts reported no cases. This phase is preceded and followed by two infection waves, respectively from March–April and October–November 2020, during which more than 1,200 cases per week were observed. Hence, we try to build a model that can adequately predict high numbers as well as zero observations, which are common during low-infection seasons, especially if one looks at granular age groups and spatial units. This is achieved by assuming that the cases follow a mixture distribution of a point mass distribution at zero and an arbitrary count distribution with mixture weight $\pi \in [0,1]$. Here, we regard any probability distribution defined over non-negative integers as a count distribution, e.g., the negative binomial, Poisson, and generalized Hermite distribution^58^. The resulting *zero-inflated* distribution $\mathcal {D}$ is primarily characterized by the mean of the count component $\lambda$ and the zero-inflation probability $\pi$. Any additional parameters relating to other traits of the count distribution, e.g., the scale parameter of the negative binomial distribution, are denoted by $\chi$. The probability mass function of $\mathcal {D}$ evaluated at *y* is given by:

9 $$\begin{aligned} f_{\mathcal {D}}(y |\lambda , \pi , \chi ) = \pi I(y = 0) + (1-\pi ) f_{\mathcal {C}}(y|\lambda ,\chi ), \end{aligned}$$

where $f_{\mathcal {C}}$ is the density of the chosen count distribution $\mathcal {C}$ and *I* denotes the indicator function. By incorporating the point mass distribution, the model can capture excess rates of zero observations and $\pi$ may be interpreted as the percentage of excess-zero observation^59^.

In our modeling approach for COVID-19 cases $y_{ig}(t)$ we relate structured data as well as network data to the parameters $\lambda$ and $\pi$ of the zero-inflated distribution, which yields the following distributional and structural assumption:

10 $$\begin{aligned} \begin{aligned} y_{ig}(t) \sim \mathcal {D}(\lambda _{ig}(t),\pi _{ig}(t), \chi ),\\ \lambda _{ig}(t) = h_1\left( \eta _{1,ig}(t)\right) ,\,\, \pi _{ig}(t) = h_2(\eta _{2,ig}(t)), \end{aligned} \end{aligned}$$

with chosen zero-inflated distribution $\mathcal {D}$. For the structural component in Eq. (10), the two feature-dependent distributional parameters are described through the additive predictors $\eta _{1,ig}(t)$ and $\eta _{2,ig}(t)$. We transform these predictors via fixed transformation functions $h_1,h_2$ to guarantee correct domains of the respective modeled parameter, e.g., a sigmoid function for the probability $\pi _{ig}(t)$.

#### Additive predictors

Inspired by SDDR^49^, additive effects of tabular features on the parameters characterizing the zero-inflated distribution are estimated using a single-neuron hidden layer. As proposed in various statistical COVID-19 modeling approaches^19,60^, these structured effects are learned with an appropriate regularization to enforce smoothness of non-linear effects. This penalization can be seen as a trade-off between complexity and interpretability^61^.

The additive predictors $\eta _{1,ig}(t)$ and $\eta _{2,ig}(t)$ can be defined in terms of both unstructured and structured features (left and right bottom input in Fig. 2). In the structured model part, we use the complete suite of district-specific features as arbitrary additive effects, detailed in the next section. In the following, we make this clear by using $\varvec{z}_{ig}(t)$, which are the input features $\varvec{x}_{ig}(t)$ but transformed using some basis function evaluation. We denote the corresponding feature weights by $\varvec{\vartheta }^{{\mathrm{str}}} = ({\varvec{\vartheta }_1^{{\mathrm{str}}}}^\top ,{\varvec{\vartheta }_2^{{\mathrm{str}}}}^\top )^\top$ corresponding to $\eta _1$ and $\eta _2$, respectively. The unstructured part of the network computes each district’s embedding (node) by exploiting time-constant district population attributes and edge attributes. For the application, these attributes encode geographic connectedness between districts and social connectedness. Here, the message passing framework enables the embeddings to contain first, second, and higher-order information about the spread of the disease among all districts. Finally, either additive predictor is augmented with the embeddings $\varvec{u}_i$ for district *i* learned from the GNN in order to incorporate the network data in the distributional framework.

#### Orthogonalization

Identifiability is crucial to the analysis at hand since some feature information is shared between the structured effects and unstructured effects. For instance, the social connectedness index $x^S$ is exploited in the structured part via the MDS-embeddings but also in the graph neural network as an edge attribute. If these two model parts are not adequately disentangled, it is unclear what part of the model is accounting for which information in the shared features. Therefore, the latent GNN representations $\varvec{u}_i$ are orthogonalized with respect to $\varvec{z}_{ig}(t)$ yielding $\tilde{\varvec{u}}_i = \varvec{u}_i \otimes \varvec{z}_{ig}(t)$, i.e., $\varvec{u}_i$ is projected on the orthogonal complement of the column space spanned by $\varvec{z}_{ig}(t)$. The additive predictors will thus use $\tilde{\varvec{u}}_i$ instead of $\varvec{u}_i$ (see^49^ for further details). In the final step, structured and unstructured effects are combined as a sum of linear orthogonalized embedding effects and the structured predictors, i.e., $\eta _{k,ig} = \varvec{x}_{ig}(t) \varvec{\vartheta }_k^{{\mathrm{str}}} + \tilde{\varvec{u}}_k \varvec{\vartheta }_k^{{\mathrm{unstr}}}$, $k \in \{1,2\}$.

#### Different exposures per district

As each district is subject to a different exposure of COVID-19, the primary goal is to model the rates of infections rather than the raw observed counts. In order to do so, the mean of the counting distribution in Eq. (10) is corrected for the differing population sizes by adding a constant offset term to the concatenated linear predictor, i.e., by adding $\log (pop_{ig})$ to $\eta _{1,ig}(t)$. For additional information on this procedure in the realm of zero inflated models, we refer to^62^.

### Proposed COVID-19 model specification

#### Distributional regression

On the basis of Eq. (10), the proposed COVID-19 model is set up as follows: let $\mathcal {D}$ define a zero-inflated Poisson (ZIP) distribution, which is reparameterized in terms of only one additive predictor $\eta _{ig}(t) = \eta _{1,ig}(t)$ as this proved to guarantee numerical stability. Therefore, define $\pi _{ig}(t) \equiv \exp \left\{ -\exp \left\{ \chi + \log (\lambda _{ig}(t))\right\} \right\}$ and the distribution’s rate as $\lambda _{ig}(t) = \exp \{\eta _{ig}(t)\}$.

Another option for modeling counts is to use a negative binomial (NB) distribution as, e.g., done in^63^. The NB distribution is often chosen over the Poisson distribution due to its greater flexibility, particularly by allowing to account for overdispersion. We will compare the NB distribution against the ZIP approach by reparameterizing the NB distribution in terms of its mean, similarly to the ZIP’s parameterization.

**Table 1:** Features and their incorporation into the structured and GNN part of the proposed model. For each feature, the second column indicates the basis function evaluation used in the structured part, which is applied to the the feature itself or a transformation of it given in brackets. If no transformation is given, the identity is used. Bivariate thin plate (TP) regression splines are used to model bivariate effects. The logp1 transformation is given by $\text{ logp1 }(y) = \log (y + 1)$. The third column indicates the incorporation of each feature in the GNN part, either as a node or edge feature. To also account for the group-specific nature of each distributional parameter, gender and age effects are added using *g* as a dummy variable

Feature	Defintion	Structured Part	GNN	
		Basis Evaluation (Transformation)	Node	Edge
$\tilde{y}_{ig}(t-1)$	Previous infections	TP-Spline ($\text{ logp1 }(\cdot )$)		
$\tilde{x}_i^G(t)$	Co-location Gini index	Bivariate Spline with *t*		
$x^S$	SCI network			$\checkmark$
$x_i^S$	MDS SCI-coordinates	Bivariate Spline		
$x^D$	Distance network			$\checkmark$
$x^N$	Neighborhood network		$\checkmark$	
$\tilde{x}_i^{SP}(t)$	Staying-put	Bivariate Spline with *t*		
$pop_{ig}$	Population	Offset ($\log (\cdot )$)	$\checkmark$	
$den_{ig}$	Density		$\checkmark$	
*g*	Gender/age group	Dummy Variables		

#### Graph neural network

Among all possible variants of the general message passing framework described above, we opt for the proposition of^64^. As a result, we can make use of multivariate edge attributes and efficiently handle relatively large graphs. The message function in Eq. (5) defined by:

11 $$\begin{aligned} M_r(\varvec{u}_v^r, \varvec{u}_w^r, \varvec{u}_{vw}) =\frac{1}{H^r\cdot |N(v)|} \sum _{h=1}^{H^r} w_h^r(\varvec{u}_{vw}) \cdot \varvec{\Theta }_h^r \varvec{u}_w^r, \end{aligned}$$

which uses $H^r$ linear maps $\varvec{\Theta }_h^r\in \mathbb {R}^{d^m_r}\times \mathbb {R}^{d^v_r}, 1\le h\le H^r$ to transform the neighbors’ features and $H^r$ radial basis function (RBF) kernels $w^r_h:\mathbb {R}^{d^v_r}\rightarrow \mathbb {R}, 1\le h\le H^r$ to weight the linear maps:

12 $$\begin{aligned} w_h^r(\varvec{u}_{vw})=\exp \left\{ -\frac{1}{2} (\varvec{u}_{vw}-\varvec{\mu }_h^r)^\top \text {diag}({\varvec{\sigma }_h^r}^2)^{-1}(\varvec{u}_{vw}-\varvec{\mu }_h^r)\right\} . \end{aligned}$$

The node update function of Eq. (6), on the other hand, becomes:

13 $$\begin{aligned} U_r(\varvec{u}_v^r, \varvec{m}_v^r)=\varvec{m}_v^r+\varvec{\Theta }_0^r\varvec{u}_v^r+\varvec{b}^r \end{aligned}$$

with trainable parameters $\varvec{\Theta }^r_0,\varvec{\Theta }_h^r,\varvec{\mu }_h^r$, $\varvec{\sigma }_h^r,\varvec{b}^r, 1\le h\le H^r, 1\le r\le R$.

Because travel is possible from any district to any other district, e.g., via train or car, we use a fully connected graph as input to the GNN and embed information about social connectedness in the edge attributes. We use two message passing rounds, i.e., $R=2$, with the first graph convolutional layer using $H^1=8$ affine maps with output dimensionality $d^v_2=256$, followed by a the second layer that further reduces this number to $d^v_3=128$ latent components with $H^2=4$. Next, four fully-connected layers successively reduce the dimensionality of the node embeddings to 16 components. All layers use batch normalization^65^ followed by leaky ReLU activation^66^. To reduce the chance of overfitting, dropout^67^ with probability 0.25 is used after the two graph convolutions. Table 1 further summarizes the use of all available features in the model using their transformation and incorporation in the structured additive as well as GNN model part.

### Uncertainty quantification

A crucial tool to investigate the model’s reliability is to assess its uncertainty. While the proposed approach explicitly models the uncertainty in the given data distribution (aleatoric uncertainty), the epistemic uncertainty of parts of the model can be derived through its connection to statistical models.

#### Epistemic uncertainty

Standard regression model theory allows to derive the epistemic uncertainty of the proposed model, i.e., the uncertainty of model’s weights. When regarding the GNN part of the model as a fixed offset $\varvec{o}$ and fixing the amount of smoothness defined by $\varvec{\xi }$ , it follows

14 $$\begin{aligned} \varvec{\vartheta }^{{\mathrm{str}}} \mid \varvec{y}, \varvec{\xi }, \varvec{o} \sim \mathcal {N}(\hat{\varvec{\vartheta }}, (\hat{\varvec{\mathscr {I}}}^{{\mathrm{str}}} + \varvec{P})^{-1}), \end{aligned}$$

where $\hat{\varvec{\mathscr {I}}}^{{\mathrm{str}}}$ is the Hessian of the negative log-likelihood at the estimated network parameters $\hat{\varvec{\vartheta }}$^44^ . We note that especially the conditioning on $\varvec{o}$ (the GNN) neglects some additional variance in the parameter estimates but still allows us to get a feeling for the network’s uncertainty.

Deep ensembles^68^ are a simple method that provides reliable uncertainty estimates, and are used to account for the epistemic uncertainty of the GNN. In this context, the epistemic uncertainty is estimated by computing the standard error of the predictions of an ensemble of models, each trained from scratch with a different random initialization.

#### Aleatoric uncertainty

In addition to epistemic uncertainty, one can account for aleatoric uncertainty by modeling all distributional parameters of the zero-inflated count distribution explicitly. For example, in the case of the ZIP distribution, the distribution’s variance can be derived from its parameters as follows:

15 $$\begin{aligned} (1 - \pi _{ig}(t)) \cdot (\lambda _{ig}^2(t) + \lambda _{ig}(t) \chi ) - (1 - \pi _{ig}(t)) \cdot \lambda _{ig}(t)^2. \end{aligned}$$

In particular, this allows us to make a probabilistic forecast for all weeks, districts, and each cohort (age, gender). After having observed the forecasted values, we can assess how well the model performed and how well it predicted uncertainty of the data distribution when only trained with historic data up to a certain week.

### Network training

Learning the parameters of the proposed model is achieved by minimizing the negative log-likelihood derived from the distributional assumption in Eq. (10). The combined weights $\varvec{\vartheta } \in \mathbb {R}^{p_1 + p_2 + \tau + p_\chi }$ of the whole network subsume $p_1$ weights for the first additive predictor $\eta _1$, $p_2$ effects for the second additive predictor $\eta _2$, $\tau$ effects for the GNN and $p_\chi$ weights for additional distributional parameters. For readability, we omit in the following the indices *i* and *g* as well as the time-dependency of the two distributional parameters, yet make the dependency on learned weights explicit. Stemming from Eq. (9), the joint likelihood $\ell (\varvec{\vartheta })$ is derived by summing up the contribution of each individual observation, that is given by

16 $$\begin{aligned} \ell (\varvec{\vartheta };y) = \log \bigg (\pi (\varvec{\vartheta }) I(y = 0) + (1-\pi ) f_{\mathcal {C}}(y|\lambda (\varvec{\vartheta }),\chi )\bigg ). \end{aligned}$$

Under conditional independence, feature weights can be learned by minimizing the sum of negative log-likelihood contributions for all observations. To avoid overfitting and help estimate smooth additive effects in the structured part of the neural network, a quadratic penalty term $J(\varvec{\vartheta }) = \sum _{j=1}^2 {\varvec{\vartheta }_j^{{\mathrm{str}}}}^\top \varvec{P}_j \varvec{\vartheta }_j^{{\mathrm{str}}}$ is used. Thereby, weights in the network $\varvec{\vartheta }_j \in \mathbb {R}^{p_j}$ that correspond to smooth structured effects are regularized. Penalization is controlled by individual complexity parameters that are incorporated in the penalty matrices $\varvec{P}_j \in \mathbb {R}^{p_j \times p_j}, j=1,\ldots ,2$. These matrices, in turn, are block-diagonal matrices that are derived by the chosen basis functions in the structured model part^44^. We do not additionally penalize the count parameter $\chi$ nor the GNN model part other than using the orthogonalization. In practice, we observe that training the network can be stabilized when choosing RMSprop^69^ as the optimizer.

## Results

**Figure 3:**
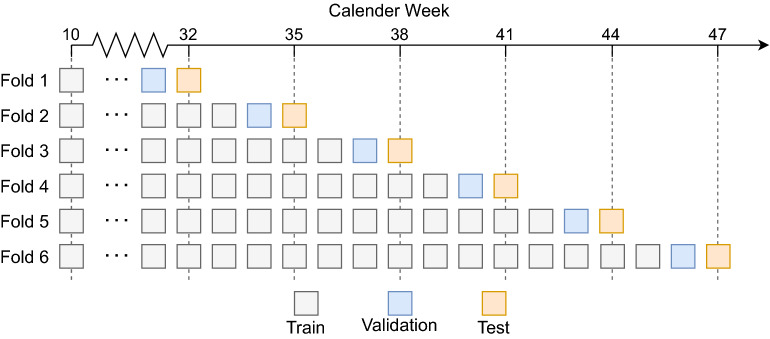
Evaluation Scheme based on an expanding window approach over the available historical weekly data

Apart from the primary goal to provide one-week forecasts, one can investigate the approach’s behavior throughout the pandemic. Therefore, we apply an expanding window approach, where we use a certain amount of data from past weeks, validate the model on the current week, and forecast the upcoming week. We do this for different time points, starting with training until calendar week 30 of 2020, validation on week 31, and testing on week 32. In 3-week-steps, we expand the time window while adapting the validation and test week. In Fig. 3, we sketch a visual representation of this scheme.

### Model comparisons

We compare the approach of this paper against four other algorithms and different model specifications of the proposed framework. As a baseline model, we use the mean of a sliding window approach applied to the given training data set (MEAN). The prediction on the test set then corresponds to the mean of the last week in the training data for each of the four subgroups (age, gender). As a statistical regression baseline we use a generalized additive model (GAM) inspired by^19^, modeling the mean of a negative binomial distribution using various smooth predictors as well as tensor-product splines. We further apply gradient boosting trees as a state-of-the-art machine learning baseline. Due to its computational efficiency, scalability and predictive performance, we chose XGBoost^70^ in the benchmark study. Finally, we compare the proposed neural network architecture against a vanilla deep neural network (DNN), a multi-layer perceptron with a maximum amount of 4 hidden layers with ReLU or tanh activation function, dropout layers in between and a final single output unit with linear activation. To enable a meaningful comparison, we corrected all benchmark model outputs for the differing exposures by incorporating an offset in same way explained previously. Similar to classical statistical models, this allows the model to learn the actual rate of infections. In all cases, we optimize the model using the Poisson log-likelihood (count loss). We furthermore tune the DNN and XGBoost model using Bayesian optimization^71^ with 300 initial sampled values for the set of tuning parameters and ten further epochs, each with 30 sampled values. Finally, we investigate the performance of a simple GNN, i.e., not in combination with distributional additive regression, optimized on the root mean squared error (RMSE).

### Forecasting performance

Table 2 shows the forecast performances of all approaches in the benchmark study. Out of the benchmark models, the GAM is the best performing models returning consistently smaller RMSE values than XGBoost and the DNN, with one exception in week 41. The rolling mean (MEAN) performs similar well before the second wave in Germany (Week 32 and 35). However, the numbers stagnate during Germany’s second lockdown (Week 47), which may be due to an external shock that cannot be accounted for by the previous weeks’ rising numbers. The vanilla DNN yields the worst performance, where the bayesian optimization found the smallest architecture with only one hidden layer with one unit to be the best option. While this result aligns with the good performance of MEAN and GAM, dropout in the DNN between the input and hidden layers does apparently not yield enough or the appropriate regularization to prevent the DNN from overfitting. The proposed model shows similar performance to the GAM model, which is again in line with what we expected, as we orthogonalize the GNN part of the network w.r.t. the whole structured additive part. In particular, the proposed model performs notably better for the weeks 41–47 than the GAM, and yields the best or second best results compared to all other models on each fold. Although XGBoost is best week 41, its worse performance on all other folds does not make it a reasonable choice. The same holds for the NB variation of the proposed approach, which overall delivers the second-best performances. Finally, the GNN itself yields reasonable predictions in the first two weeks but does not perform well for the other weeks.

**Table 2:** Root mean squared error values for different methods (rows) and folds (columns) Bold numbers denote the best result in each fold across all models

	Calendar Week					
	32	35	38	41	44	47
XGBoost	4.926	5.188	7.447	**15.327**	65.036	74.235
DNN	10.179	12.178	17.897	64.065	108.474	80.901
GAM	4.042	4.738	4.736	21.666	18.556	23.813
MEAN	5.038	**3.666**	6.196	30.910	20.090	23.159
GNN	5.972	6.785	11.355	49.064	77.162	53.489
Ours (ZIP)	**3.931**	4.235	**4.500**	16.588	**17.738**	**15.050**
Ours (NB)	4.096	4.094	5.174	28.580	18.098	31.724

### Model interpretation

**Figure 4:**
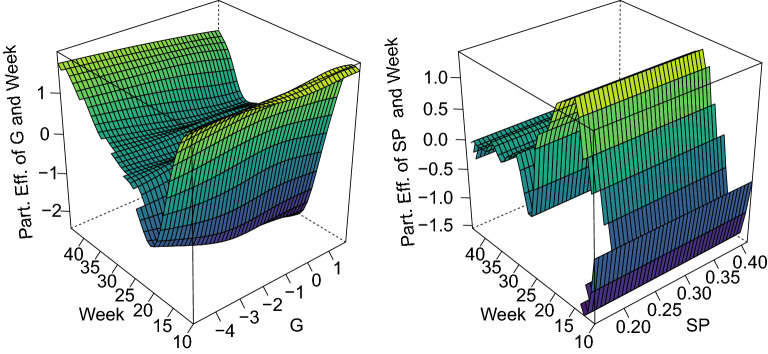
Estimated bivariate partial effects of the week and Gini coefficient (G) on the left as well as of the week and Percentage Staying Put (SP) on the right. For weeks 10 to 30, low standardized Gini coefficients come with low infection rates. Thus, more focused meeting patterns are associated with lower infection rates in that time frame. The effect of people staying put is negative at the beginning of the observational period and in the end. Hence, a higher percentage of people staying put is related to lower infection rates

To begin, we investigate the partial effects of the Gini coefficient (G) derived from the colocation maps and the percentage of people staying put (SP) on the left and right side of Fig. 4, respectively. Moreover, a high standardized Gini coefficient translates to meeting behavior that is more dispersed than the average of all districts. Hence, a low standardized Gini coefficient (less mobility than average) leads to lower infection rates, especially between calendar week 10 and 30. For the percentage of people staying put, the temporal dynamics are somewhat opposite and exhibit small effects in the first weeks and after week 30. Thereby, we may conclude that having a higher percentage of people staying put also lowers the infection rates. Further, we observe that the incorporated penalty term successfully regularizes the bivariate effect term to be a linear effect in the direction of the percentage of people staying put.

### Epistemic uncertainty

**Figure 5:**
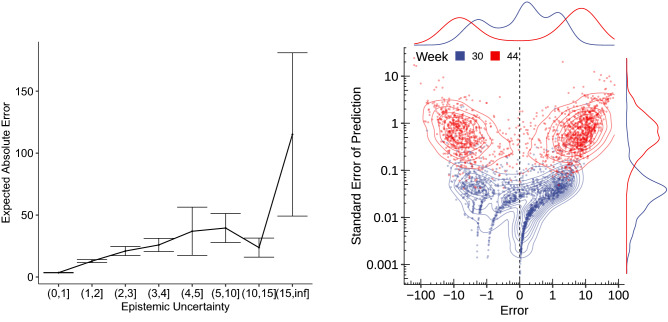
Left: average absolute error incurred by the ensemble for increasing levels of epistemic uncertainty, with vertical bars denoting ± one standard error of the estimation. Right: Standard error of the predictions of an ensemble of ten networks correlated with the error incurred when using the ensemble’s mean prediction for each district, age, and gender cohort during a low-infection phase (week 30) and the second wave of infections (week 44)

While an epistemic uncertainty for the structured part of the proposed model can be derived theoretically, the models’ uncertainty is estimated through an ensemble for the GNN part as detailed in the previous section. More specifically, we focus on two instances of the proposed models that are trained with data until calendar week 29 and 43, and evaluate on following weeks 30 and 44. These weeks were chosen to showcase the effects of uncertainty during a high and low season of the pandemic.

The epistemic uncertainty is well correlated (Spearman’s $\rho =0.76$) with the absolute error resulting from the mean prediction (Fig. 5 left) and grows approximately linearly with the error. However, the variability of the average error is not reliable in the last bin as it only contained four samples. The epistemic uncertainty is generally larger for high-incidence weeks such as week 44 when compared to a low-incidence week such as week 30 (Fig. 5 right). In addition, the ensemble has a very slight tendency to underestimate the number of cases for week 44 by 1.26, and to overestimate the cases for week 30 by 0.25. Although statistically significant (one-sided *t*-test, $t=3.24$, $p=0.001$ and $t=3.38$, $p=0.0007$, respectively), the resulting differences are practically irrelevant, hence suggesting that the ensemble is approximately well-calibrated. In general, this correlation between epistemic uncertainty and forecast error provides a reliable diagnostic of the trustworthiness of the proposed model’s predictions.

**Figure 6:**
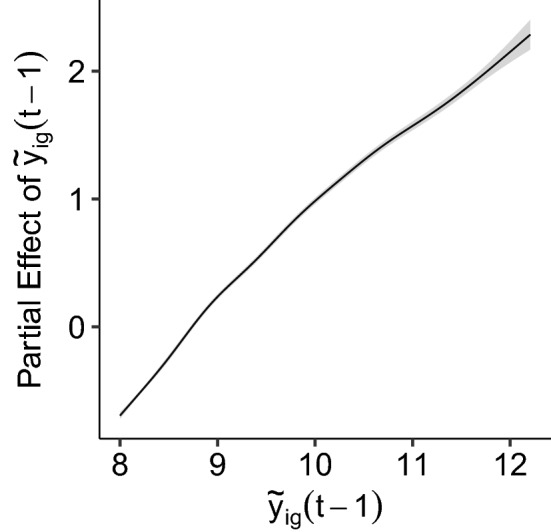
Estimated effect of lagged infection rate with corresponding confidence intervals for week week 44

The partial effect of lagged infection rates in Fig. 6 additionally depicts its epistemic uncertainty when the GNN weights are fixed. The figure’s narrow shaded areas translate to high certainty of the partial effect from the respective feature. Moreover, the partial effect translates to the finding that the higher the infection rate was in the previous week, the higher the predictions are in the upcoming week. This result alignes well with prior studies, that identified this feature as a principal driver of infection rates^19^.

### Aleatoric uncertainty

**Figure 7:**
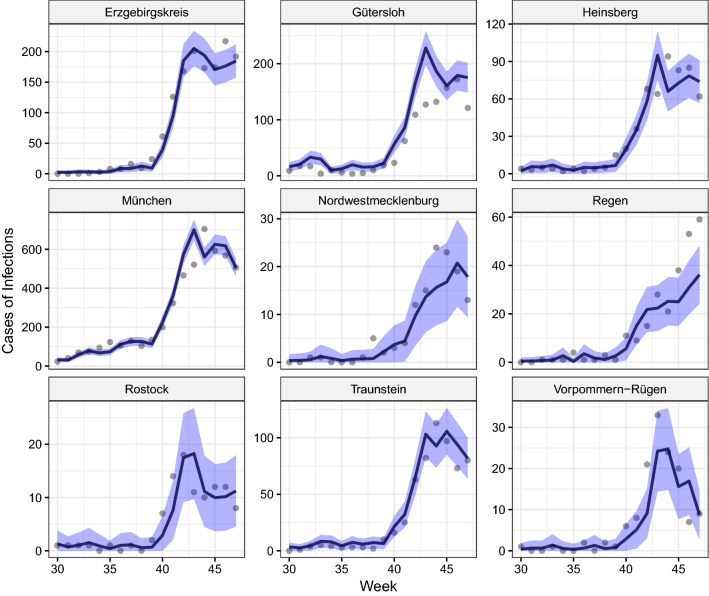
Exemplary prediction intervals for age group 35–59 and gender male (results of other groups can be found in the Appendix) and selected districts (facets) in Germany. For the different forecast weeks (x-axis) the true number of infections are visualized by points and contrasted with the model’s prediction (black line) and the prediction interval (shaded purple area)

**Figure 8:**
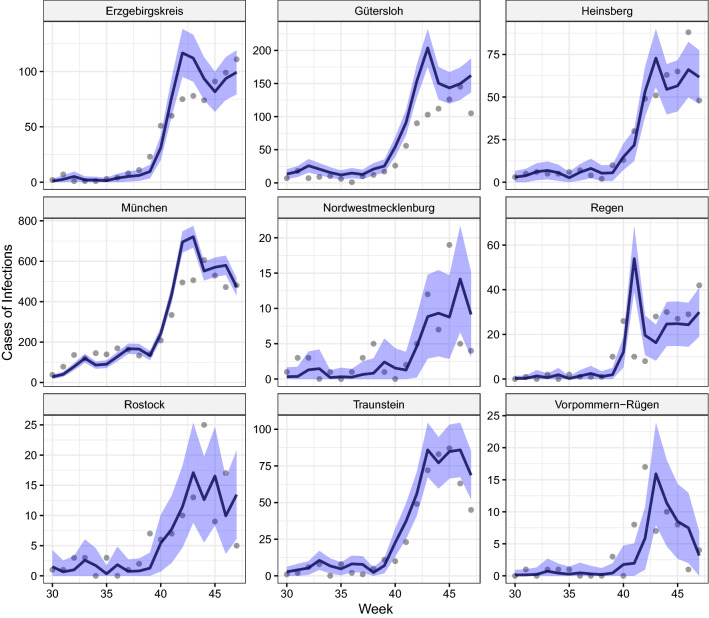
Prediction intervals for age group 15–34 and gender male and selected districts (facets) in Germany. For the different forecast weeks (x-axis) the true number of infections are visualized by points and contrasted with the model’s prediction (black line) and the prediction interval (shaded purple area)

**Figure 9:**
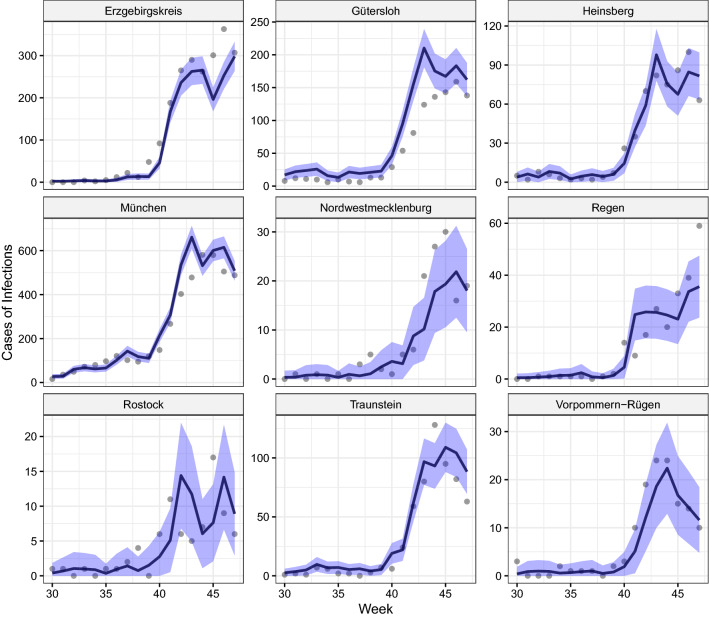
Prediction intervals for age group 35–59 and gender female and selected districts (facets) in Germany. For the different forecast weeks (x-axis) the true number of infections are visualized by points and contrasted with the model’s prediction (black line) and the prediction interval (shaded purple area)

**Figure 10:**
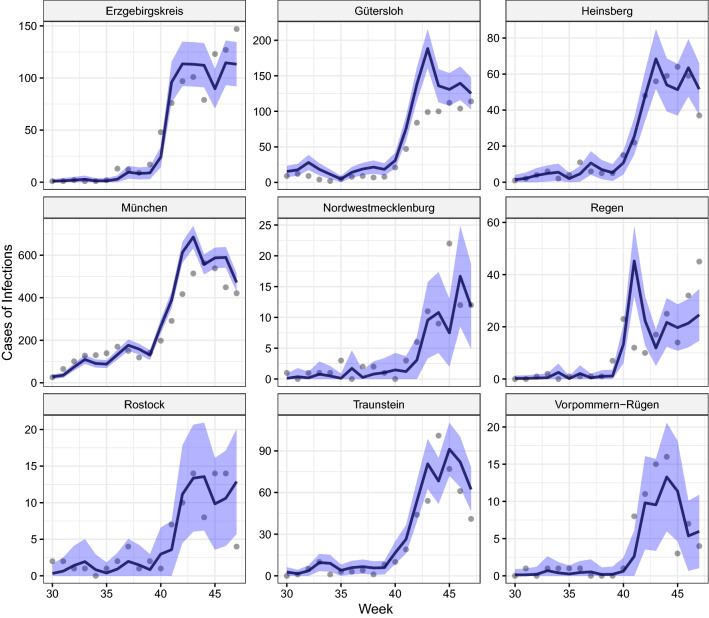
Prediction intervals for age group 35–59 and gender female and selected districts (facets) in Germany. For the different forecast weeks (x-axis) the true number of infections are visualized by points and contrasted with the model’s prediction (black line) and the prediction interval (shaded purple area)

We evaluate the ZIP model’s aleatoric uncertainty by applying the expanding window training scheme analogous to the previous evaluation. For each prediction, we calculate predictive distribution intervals using the mean prediction ± 2 times the standard deviation derived from Eq. (15). Figure 7 depicts the probabilistic forecasts of the modeled ZIP distribution for different districts in Germany. These districts constitute particularly difficult examples from relatively rural areas and cases in larger cities such as *München* as well as sites that were hardly and severely affected by the pandemic. Figure 7 visualizes the true values as points and the predicted mean as a black line. Here, the shaded purple area symbolizes the predictive distribution intervals. We observe that most of the points are well within the given prediction interval, thus the distribution captures the dispersion in the data quite well. As expected from the Poisson distribution, results indicate that the aleatoric uncertainty increases with the rising number of infections. However, some intervals are not able to cover larger fluctuations and steeper increases of infections, such as is the case in *München*, *Gütersloh* or *Vorpommern-Rügen*. While Fig. 7 showcases the predictions for the age group 35–59 and gender male, results for other groups can be found in Figs. 8, 9, 10 in the Appendix.

Overall, the intervals derived from the predictive distributions cover on average over 80% of all cases in all groups, weeks and districts. This indicates that the estimation of distribution variances for groups and weeks works well, but shows also room for improvement for later weeks where the distribution is not perfectly calibrated.

## Discussion

Following several experts’ call to account for human mobility in existing statistical and epidemiological models of COVID-19, we proposed a multimodal network that fuses existing efforts with graph neural networks. We thereby enable the use of a more nuanced collection of data types, including mobility flows and colocation probabilities, in the forecasting setting.

### Results

The results indicate a notable improvement over existing approaches, which we achieved by incorporating the network data. The provided findings also highlight the need for regularization and showcase how common ML approaches can not adequately capture the autoregressive term, which, in turn, proved to be essential for the forecast. The proposed model’s investigation further reveals that uncertainty can be well captured by the model, although further calibration may be vital for its aleatoric uncertainty. We also conclude that the proposed model captures measures of social distancing by the German government. Figure 4, e.g., shows that an increase in the Gini index (a decrease in mobility) results in lower predicted incidences. Due to the nature of the data, we can, however, not draw any causal conclusions as public health policies apply for all residents in Germany.

### Caveat

We also want to emphasize that despite the convincing results, the given analysis only addresses a small subset of processes involved in the spread of COVID-19 and should not be the sole basis for decision-making processes in the future. In particular, forecasting infection rates in the short run does not (need to) address reporting or observation biases typically present for such data and requires a solid data basis, which the Robert–Koch Institute provides for Germany. Furthermore, working only with mobility data from one source, i.e., Facebook, might affect the findings due to an unknown selection bias. To minimize such a bias, we restricted the analysis to the younger and mid-aged cohorts. Furthermore^19^, investigate the representativeness of the Facebook data and compare the information with the mobility data from other providers, such as Google and Apple, and conclude that the data source adequately represents the spatial distribution of people and is consistent with the other data sources.

### Future work

In light of the positive results presented in this work, as well as its present limitations, we can foresee several research avenues to improve spatio-temporal models further. First, using time-varying, as opposed to static networks, either through auto-regressive or recurrent architectures, would enable direct modeling and exploitation of the time dynamics of human mobility.Second, the semi-structured approach of this article could be extended to incorporate epidemiological models such as SIR as a third additive predictor. Finally, additional data sources could be used, possibly of higher granularity, e.g., daily instead of weekly infection count, to provide faster and more accurate insights into the pandemic.

## Conclusion

Reliable and interpretable forecasts of disease incidents, especially accounting for human mobility, are a vital tool to enable policymakers to manage healthcare resources in the optimal way. We proposed a novel multimodal approach that combines network-valued and spatio-temporal disease data in an interpretable manner and provides the best predictive performance compared to traditional statistical, machine and deep learning methods.

## Further results

Figures 8, 9, 10 visualize the prediction intervals for the other combinations of age and gender. Plots show little difference to Fig. 7 both in terms of actual numbers of cases and in terms of the generated prediction intervals.

## Data for good

For this project, we used data provided by Facebook through the *Data for Good* program. The data is in parts openly accessible. From this open-access data, we use the social connectedness index (which can be accessed under https://dataforgood.facebook.com/dfg/tools/social-connectedness-index#accessdata and the movement range maps (https://dataforgood.facebook.com/dfg/tools/movement-range-maps). Additionally, we employ the Co-location networks detailed under https://dataforgood.facebook.com/dfg/tools/colocation-maps. This data source is not directly openly available, but can be accessed after signing a data agreement form at https://dataforgood.facebook.com/dfg/tools/colocation-maps#accessdata.
